# Economic vulnerabilities, mental health, and coping strategies among Tanzanian youth during COVID-19

**DOI:** 10.1186/s12889-024-18074-z

**Published:** 2024-02-22

**Authors:** Stephanie Simmons Zuilkowski, Sarah Quinones, Hassan Kihanzah, Graca Marwerwe, Leah Prencipe, Lusajo Kajula, Tia Palermo

**Affiliations:** 1 Educational Leadership and Policy Studies Learning Systems Institute University Center C4600, Florida State University, Tallahassee, FL USA; 2 Department of Epidemiology and Environmental Health, Division of Health Services Policy and Practice, University at Buffalo (State University of New York) , Buffalo, NY USA; 3 Independent Researcher , and University at Buffalo (State University of New York) , Dar es Salaam, Tanzania; 4 Department of Social Epidemiology, Erasmus MC, Rotterdam, Netherlands; 5 Independent Researcher , and Muhimbili University of Health and Allied Sciences , Dar es Salaam, Tanzania

**Keywords:** Youth, COVID-19, Mental health, Tanzania, Africa

## Abstract

**Background:**

The COVID-19 pandemic has exacerbated struggles for youth living in poor households. Youth in rural Tanzania are particularly vulnerable given widespread poverty, lack of formal sector employment opportunities, and health risks. We examine influences of the pandemic on economic insecurity and mental health and explore the coping strategies employed by youth and their households.

**Methods:**

We conducted mixed-method data collection with youth (*N* = 760 quantitative and *N* = 44 qualitative interviews) and households (*n* = 542) via mobile phone among a sub-set of a cohort from an on-going longitudinal sample in two rural regions in Tanzania. In addition to phone interviews, we collected data bi-weekly via SMS messaging. We present mixed-methods, descriptive analysis of the outcomes and longitudinally compare quantitative outcomes pre- and post-COVID-19, within the same individuals.

**Results:**

Adverse economic impacts were most salient, and to cope, youth engaged in more labor and domestic chores. Compared to prior the COVID-19 pandemic, youth reported spending more time caring for elderly or sick household members and gathering firewood or nuts.

**Conclusions:**

These findings underscore the potential opportunity to promote policies and programs which address risks youth face. Recommended measures include expansion and adaptation of social protection policies, strengthened food and nutrition surveillance and referral systems, and scaling up community-based mental health programming.

**Supplementary Information:**

The online version contains supplementary material available at 10.1186/s12889-024-18074-z.

## Introduction

The Coronavirus pandemic of 2019 (COVID-19) has led to unprecedented challenges on a global scale. These include adverse health impacts among individuals, but also overburdening of health systems and economic impacts. As of September 2022, there had been more than 600 million cases of COVID-19 globally, with more than six million deaths [1]. In Tanzania, there had been 39,168 reported COVID-19 cases and reported 845 deaths [1]. However, these numbers are likely be underestimates given the limited resources for surveillance in Africa. Due to closures to prevent the transmission of COVID-19, approximately 40% of school-aged children in Eastern and Southern Africa were out of school as of July 2021, creating severe implications for future educational attainment and income potential [2]. Job losses in Africa resulting from COVID-19 ranged from 8 to 62% of the employed population across 11 countries [3]. Relatedly, COVID-19 has reduced access to food and increased food insecurity [4].

These adverse economic and schooling impacts are likely to affect youth well-being and safe transitions to adulthood. Adolescence represents a critical stage in development whereby vulnerability increases as a result of increased biological demands of the body and social demands as individuals experience transition into adulthood. Thus, individuals in this life phase may be acutely vulnerable to sudden, adverse economic shocks. For example, youth’s experiences with food insecurity may affect key transitions into adulthood as poor nutritional status can influence school attendance and achievement, employment, and overall health and well-being [5]. Moreover, school closures can affect educational attainment, with implications for other adverse outcomes, including early pregnancy and marriage and reduced future earning potential [6–8].

Low educational attainment, often a useful proxy for poverty, has also been associated with worse mental health and increased risk of violence [9]. Among youth in Africa, there are observed associations between school drop-out, child labor, and family formation [10]. Youth may leave school due to pregnancy, lack of capital for educational expenses or to help the household economic situation by engaging in income generating activities [11].

Existing research on youth and COVID-19 suggests impacts have been felt across widespread domains, including food security, reproductive health access, mental health, and violence. One study in Kenya indicated that food insecurity was reported by more than 75% of adolescents [12]. Financial insecurity also negatively impacted health care seeking among adolescents [12]. These changes in healthcare seeking behavior during COVID-19, in turn, had adverse impacts on adolescent pregnancy, as contraceptives were not sought or were not available at health facilities [13]. Similarly, studies examining impacts of the Ebola epidemic in Western Africa between 2014 and 2016 found that school closures led to increased adolescent childbearing [14]. Additionally, violence has increased during COVID-19 lockdowns and gender-based violence has been termed the “twin pandemic” as women and girls have been made more susceptible to violence and discrimination [15, 16]. During the COVID-19 pandemic, adolescent girls and young women in South Africa were found to have experienced increases in stress and anxiety in addition to fear, violence, and food insecurity [17].

However, few studies have specifically examined coping strategies (either from a mental health perspective or more broadly from a social and economic perspective), and an existing narrative review of studies on mental health and coping within the context of COVID-19 found that existing studies were largely based in Europe and Asia. The current study addresses this gap with a contribution from an African country examining trends across a broad range of mental health and economic outcomes before and during COVID-19 and coping strategies employed to deal with challenges brought on by the pandemic [18]. We examine a range of outcomes to emphasize that adolescent well-being is multidimensional, and that the various dimensions (economic security, mental health, schooling, violence and exploitation), are all interrelated and affect the transition to adulthood.

## Tanzanian context

After announcing the first COVID case on March 16 2020, the Tanzanian government banned all forms of public gatherings, including seminars, weddings, and sports activities. Schools were closed between March 16 and June 28, 2020, and international flights were cancelled between April 11 and May 18, 2020. The Ministry of Health, Community Development, Gender, Elderly and Children (MoHCDGEC) issued a series of Standard Operating Procedures (SOP) which detailed procedures for community-based prevention efforts, including masks for sick persons and physical distancing in the context of health care, public transport and burials. Nevertheless, social distancing and other protective measures were not as extensive and were of shorter duration in Tanzania compared to other countries. In fact, in June 2020, the late President John Magufuli declared COVID-19 no longer a threat to the country. However, one month prior to this declaration, the US embassy warned that hospitals in the commercial capital, Dar es Salaam were overwhelmed and COVID-19 risk was extremely high [19]. While the Magufuli administration denied this, after his death in March 2021, the new administration, led by President Samia Suluhu, formed a COVID-19 response committee which included prominent microbiologists and other scientists and implemented new prevention measures including COVID vaccines.

The first wave of the pandemic significantly impacted lives and livelihoods in Tanzania. Economic activity slowed, policy decisions discouraged investment, and firms faced questions regarding the viability of global supply chains [20]. Despite the short duration of prevention measures in Tanzania specifically, the global economic slowdown negatively affected tourism, exports and foreign investment. In contrast, however, Tanzanian gold exports benefited from the crisis, as gold prices rose between 2019 and 2020 [20]. Government revenues also declined due to declines in production, tourism, consumption, and imports. In 2020, the pandemic had caused the poverty headcount ratio to rise from 26.1 in 2019 to 27.2% in 2020. Then, as of February 2021, it was estimated that the pandemic could push an additional 600,000 Tanzanians into poverty, and the country’s economic outlook remained highly uncertain [20]. In turn, despite the fact that schools re-opened in July 2020, it is possible that the economic downturn may have affected households’ ability to send children to school (either due to lack of funds to purchase uniforms, books and other needed items, or due to the need to engage children in alternative activities such as agriculture or other forms of labor).

Another important contextual factor in understanding the findings from this study that our sample comes from a longitudinal study of households participating in a large-scale government social protection program, the Productive Social Safety Net (PSSN). The PSSN is comprised of a cash transfer program, public works in the lean season, and livelihoods enhancement and is targeted to the poorest 10% of households nationally. Households in this study had been enrolled in the PSSN and receiving predictable payments (variable, but up to a maximum of 18 USD, with the average payment representing 21% of pre-program household expenditures) every other month between 2015 and 2019. The program has had positive impacts on poverty reduction, food security, school enrolment, mental health, and other outcomes [21–23]. However, between March 2019 and September 2020, payments were stopped due to prolonged negotiations between the Government of the Republic of Tanzania the World Bank, which provides a majority of funds for the PSSN in the form of loans. This meant that households that depended on the PSSN for their basic needs faced a double economic shock in the form of loss of PSSN payments and the COVID-19 pandemic during the period studied.

The adverse effects on schooling, health, and economic conditions pose a serious setback to the country’s five-year development plan and its potential to harness the demographic dividend. In the next 10 years, Tanzania’s largest ever youth population will enter its productive years. This in turn can accelerate economic growth during the demographic dividend, resulting from decreasing fertility and an increased share of the working-age population compared to dependents (the young and elderly).

## Pathways of COVID impacts on youth and child well-being

**Fig. 1:**
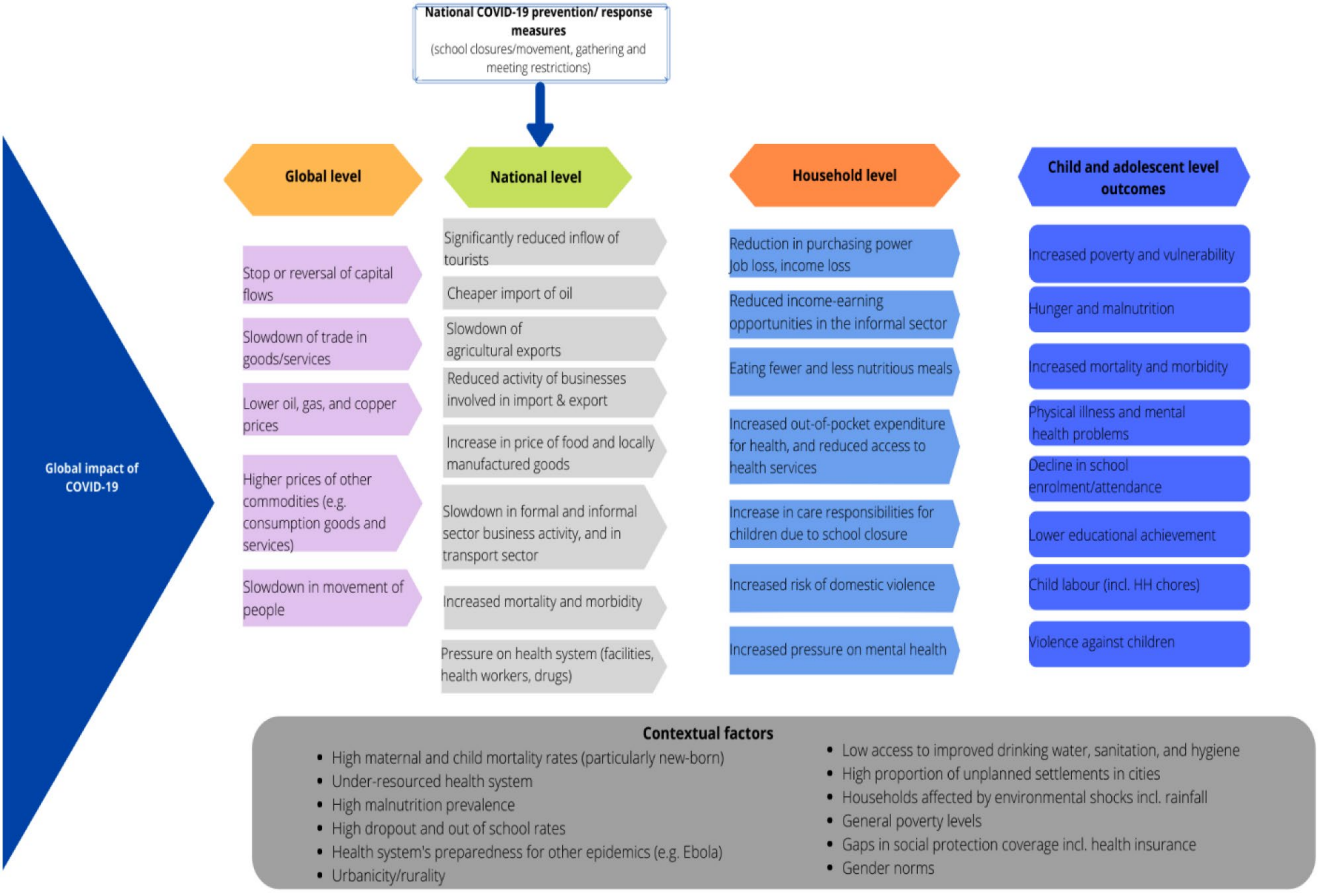
Conceptual framework

There are several pathways through which the COVID-19 pandemic may influence youth outcomes (Fig. 1). During the SARS-CoV-2 outbreak, the WHO Strategic and Technical Advisory Group for Infectious Hazards (STAG-IH) recommended a combination of responses including containment measures to delay onset of patient surges, public awareness campaigns, promotion of personal hygiene, preparation of health systems, and postponement or cancellation of large-scale public gatherings [24]. Tanzania’s compliance with some recommendations, combined with global supply chain issues, led to reduced tourism, slowdown of agricultural exports, reduced business activity, and increased prices of food and other goods.

In turn, households experienced job losses and/or income reduction, increased food insecurity, and increases in out-of-pocket health expenditures. School closures increased time children spent engaged in household chores, while women and children were at increased risk of violence. The totality of these experiences is hypothesized to have taken a serious toll on the mental health of individuals affected.

Following the immediate household-level shocks, children and youth were likely to experience increased poverty and vulnerability, hunger and malnutrition, declines in school enrollment and attendance, lower educational attainment, increased child labor (including engagement in casual labor), more depressive symptoms, and greater violence.

It is important to understand the challenges youth have faced during the pandemic globally to help mitigate these and to prevent long-term consequences of these events. It is also important to understand resiliency factors to further strengthen and support these. In the current study, we aimed to examine impacts of the COVID-19 pandemic among youth living in rural Tanzania on economic well-being and mental health and coping strategies employed in the face of these challenges. This study adds to the small existing literature on COVID-19 and youth health and well-being in the following ways: (1) we examine both male and female youth (2) data are drawn from individuals living in rural communities of Tanzania (3) we used a mixed-methods approach with both qualitative and quantitative analyses.

## Methods

### Sample

**Fig. 2:**
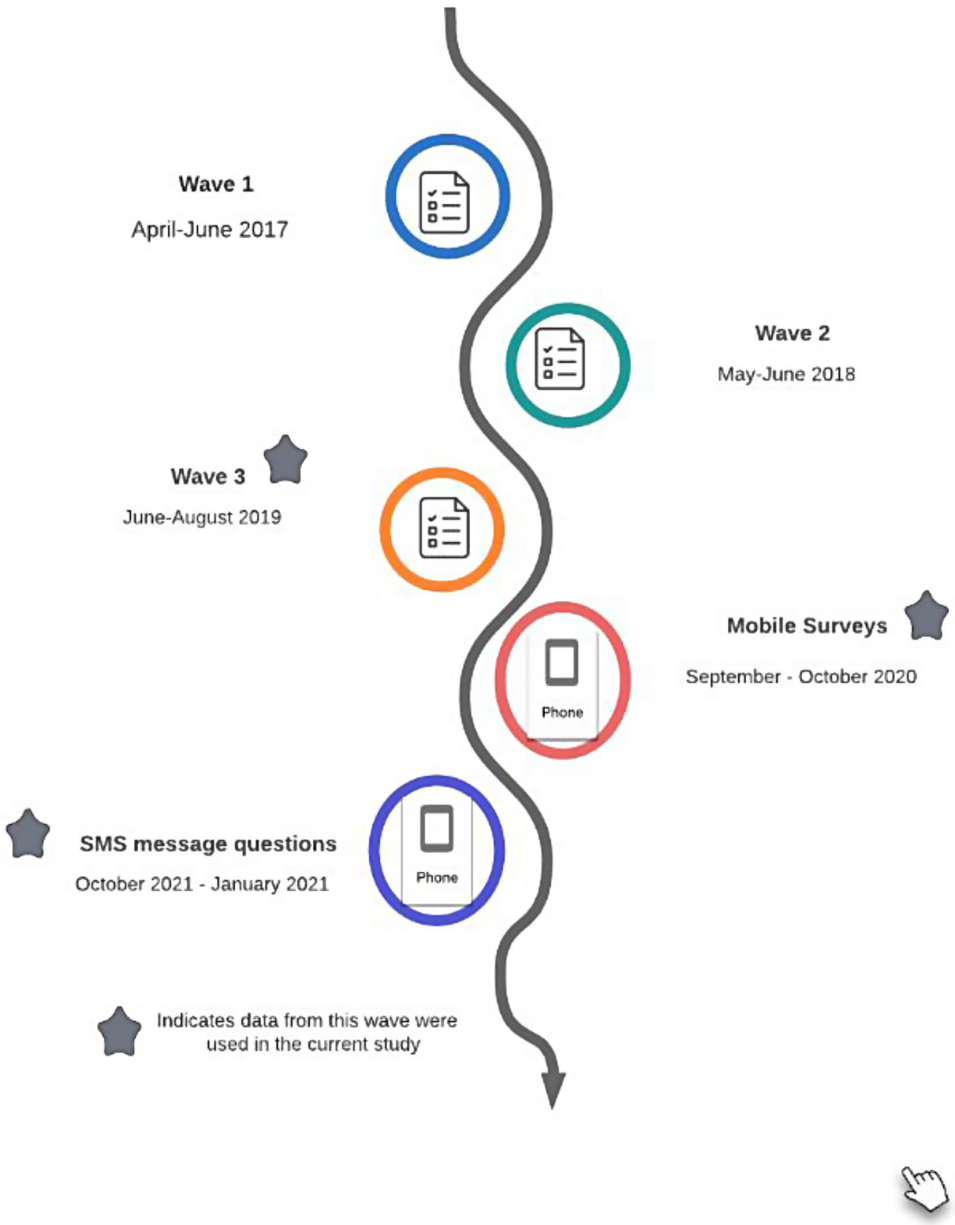
Timeline of data collection

Data used in this study come from a longitudinal impact evaluation of the “Ujana Salama: A Cash Plus Model for Safe Transitions to a Healthy and Productive Adulthood” program. Implemented between 2018 and 2019, this pilot intervention operates within the PSSN. The PSSN is comprised of cash transfers delivered every other month, combined with livelihood enhancement support and a public works program during the lean season(s). The Tanzania Social Action Fund (TASAF), in collaboration with TACAIDS and technical support from UNICEF, implemented this intervention. The broader impact evaluation was implemented between 2017 and 2021 (see Fig. 2 for timeline) in two regions of Tanzania, Iringa and Mbeya, and seeks to measure pilot impacts targeted to youth aged 14 to 19 years at baseline living in PSSN recipient households [25]. PSSN households, among the poorest 10% of Tanzanian households, began to receive program benefits in 2015. These benefits have myriad positive impacts on poverty reduction, improved food security, school attendance, and more [22, 23]. Given PSSN eligibility requirements for the pilot intervention, the sample examined in this study is a homogenous, extremely food-insecure sample of households and adolescents.

A total of 130 remote and rural villages were randomized to either treatment or control study areas within Iringa (Mufindi District Council (DC) and Mafinga Town Council (TC)) as well as Mbeya (Rungwe DC and Busokelo DC). After baseline (2017, *n* = 2,458), this sample was followed-up again in 2018 and 2019, with re-interview rates of 86% and 89%, respectively [26, 27]. In 2020, quantitative and qualitative data were collected via mobile phone surveys, which are used for the current study. The current study is observational and cross-sectional and does not examine impacts of the intervention. More detailed information on evaluation’s study design and sampling is provided in Supplementary information 1. The eligibility criteria for the current study include being part of the larger longitudinal impact evaluation; and (1) aged 18 years or older; or (2) currently married. We made no efforts to contact unmarried minors in this mobile data collection wave due to ethical concerns of securing parental consent over the phone to interview this subsample. A total of 1,727 youth were eligible for this study based on these criteria, and we aimed to sample 1,066 for the mobile quantitative surveys. We successfully completed interviews with 760 youth (71% response). Further, 44 youth were selected for semi-structured interviews (in September and October). This is a purposive sample and 2/3 were selected because they were living in households with school-aged children or were particularly vulnerable youth, including having previously experienced depressive symptoms, violence, or pregnancy.

Additionally, a subsample of 542 household heads from households where youth live were interviewed via mobile phone. Messages sent to household heads included four short questions. Further, qualitative interviews were conducted with 16 randomly selected community leaders.

Youth quantitative surveys were administered between September 7, 2020 and October 5., 2020 by EDI Global via mobile phone interviews. Consent calls with households were also conducted during this period for the SMS surveys. These consent calls were used as opportunities for the first round of data collection using SMS questionnaires but were delivered orally (heretofore referred to as “Round 0”). All interviews were conducted in Swahili. Survey responses were input into Surveybe software by enumerators while qualitative interviews were recorded, transcribed, and translated into English.

Supplementary Fig. 1 illustrates further details of the sample and timing of interviews.

### Measures

**Fig. 3:**
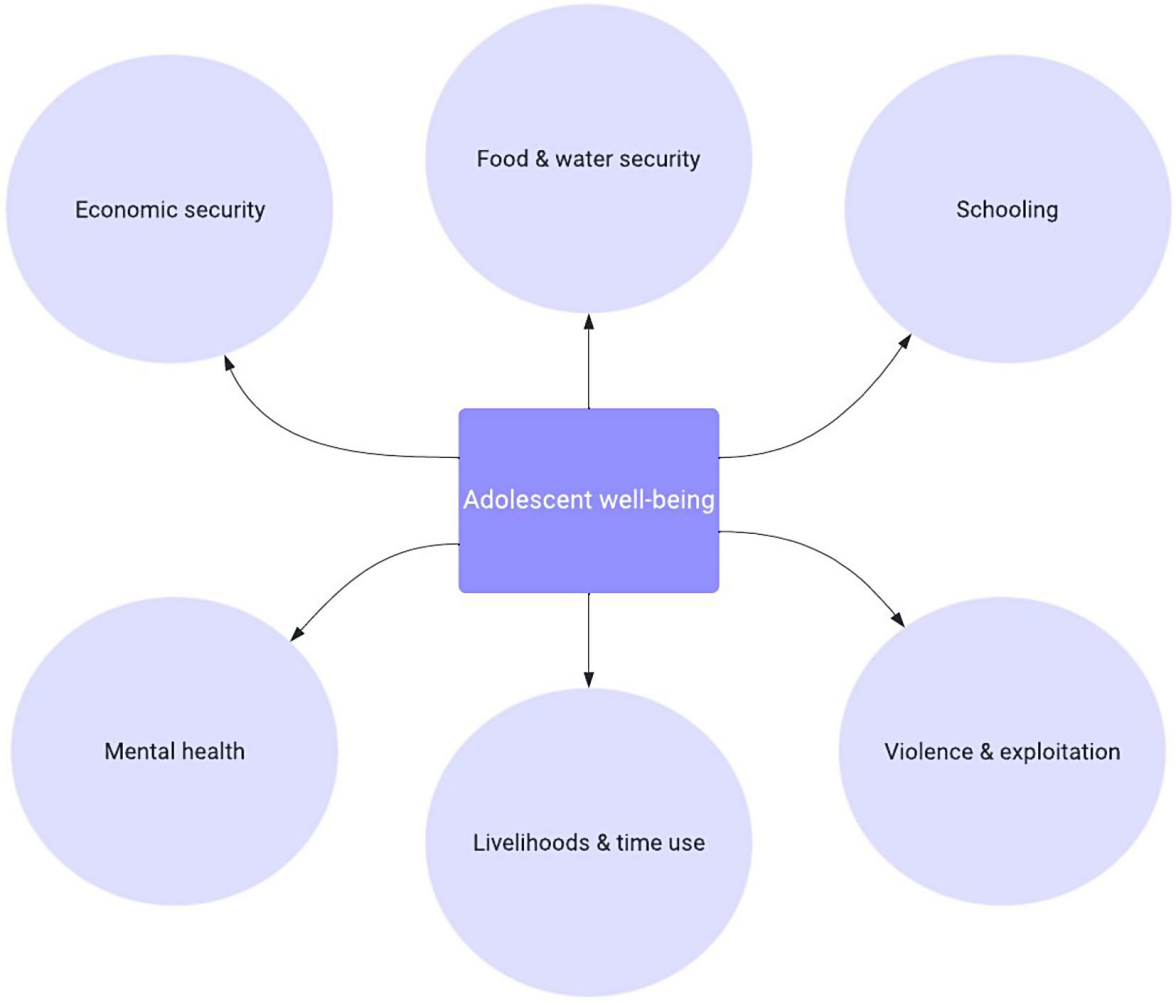
Dimensions of well-being covered in current study

This study covered a broad range of topics, all reflecting interrelated vulnerabilities in adolescence, including COVID-19 knowledge, prevention, and messaging; livelihoods and time use; food security; schooling; and violence and exploitation (see Fig. 3 for illustration of these interrelated dimensions of adolescent well-being). Below, we describe the quantitative measures and the qualitative protocols.

For livelihoods and time use, we inquired about the number of hours in the past seven days youth engaged in farm work, caring for livestock or poultry, non-agricultural business activities, or paid work for someone outside of the household. Specifically, the question asked: “During the last 7 days, how many hours did you spend in farm work, excluding livestock activities, for the households or on your own account (either in cultivating crops or in other farming tasks) on a farm owned, rented, or share cropped by yourself or a member of the household?” Then, a binary indicator was created to reflect any engagement in any of these activities if respondents reported spending any amount of hours (> 0 h) in a particular work activity. Additionally, we asked about the number of hours that youth participated in domestic chores in the past 24 h, including collecting water or firewood; collecting nuts or other tree fruits; care of children, cooking, or cleaning; and taking care of elderly or sick household members. Separately, youth were asked: “How many hours did you spend yesterday collecting water or firewood?”; “How many hours did you spend yesterday collecting nuts or other tree fruits, honey, or other products from the forest, either for food consumption, medicine, or sales for the household?”; “How many hours did you spend yesterday taking care of children, cooking or cleaning?”; and “How many hours did you spend yesterday taking care of elderly or sick household members?”

Measures of food and water insecurity were included in both youth and household quantitative surveys. Drawn from existing food insecurity questions [28], the food insecurity item included in the household survey was as follows: “In October, did you or any household member have to eat some foods that you really did not want to eat because of a lack of resources?” The two following items were included in the youth surveys: “In the past four weeks, were you or any household member not able to eat the kind of foods you preferred because of lack of resources?” and “In the past four weeks, did you or any household member have to eat some foods that you really did not want to eat because of a lack of resources to obtain other types of food?” Response options to these questions included never, rarely (1–2 times), sometimes (3–10 times), and often (> 10 times). Items were analyzed individually.

We measured water insecurity using items from the Household Water Insecurity Experiences (HWISE) Scale [29]. For this study, two items from the short-form called the HWISE-4 were used [30], including “In the last four weeks, how frequently has there not been as much water to drink as you would like for you or anyone in your household?” and “In the last four weeks, how frequently have you or anyone in your household had to go without washing hands after dirty activities (e.g., defecating or changing diapers, cleaning animal dung) because of problems with water?”. Responses to these questions included never, rarely, sometimes, often, and always.

We combined the “sometimes” and “often” responses for food and water insecurity, creating a binary indicator equal to one if a youth provided either response to these questions for the analyses.

We asked youth whether, in response to changes in general economic conditions in the past six months and the resulting hardships, they engaged in a number of coping strategies. We classified the following six strategies as “negative” coping mechanisms: changed eating patterns, sold agricultural assets, sold durable goods, sold land, sold crop stocks, and sold livestock. We then created a scale ranging from 0 to 6 indicating how many negative coping strategies youth employed.

Youth were asked if they were currently attending school. Further, we asked household heads “Has a lack of money prevented any children in your household from attending school?”. We consider whether a lack of money prevented sending children to school both for the entire sample and for the sample of households with school-aged children.

To assess mental health, we selected two items from the 10-item Centers for Epidemiological Studies-Depression Scale (CES-D10) [31]. Youth were asked the following two questions: (1) “In the past seven days, how often did you sleep well?” and (2) “In the past seven days, how often were you bothered by things that don’t usually bother you?”. Responses included rarely (< 1 day), sometimes (1–2 days), most of the time (3–4 days), and all of the time (5–7 days). We created binary indicators for each item, wherein “poor sleep” equals one if the youth reported they slept well rarely or sometimes (zero if youth slept well most or all the time) and “bothered” equals one if the youth reported being bothered sometimes, most of the time, and all of the time (zero if youth rarely bothered by things).

One item from an existing scale to measure transactional sex was used, namely, “In the past 12 months, did you start a sexual relationship with someone in order to get things that you needed, such as money or gifts?” Again, we used one item from the scale and not the full scale. The item draws on questions recommended by Wamoyi et al. (2019) [32], which was informed by the STRIVE transactional sex working group and research previously conducted in Tanzania [33–36].

To assess economic conditions, namely prices and food availability, we asked youth whether prices increased since March 2020 more than usual at the market where their household usually shops. Additionally, we asked if households were unable to purchase any food or essential items that are frequently purchased due to limited supply or unavailability in the market. Finally, we asked generally whether standard of living is worse, the same, or better compared to before March 2020.

### Qualitative interview protocol

The semi-structured qualitative interviews focused on the same topics covered by the quantitative surveys (see Supplementary File for the youth interview protocol). Interviewers used follow-up and probe questions to better understand the lived experiences of the participants.

### Analysis

A subset of eligible youth from the overall impact evaluation sample was selected due to logistical and budgetary constraints in re-contacting all youth in the cohort. To evaluate potential biases introduced by this sampling approach, we compared characteristics of youth in the full cohort to those eligible for mobile surveys and compared those eligible for mobile surveys to those interviewed for mobile surveys (Supplementary Table 1). These comparisons were made using ordinary least squares (OLS) regression with the variable of interest as the dependent variable and group (full sample, eligible, or interviewed youth) membership as the independent variable. All regression models were adjusted for district and village size and standard errors were adjusted for clustering at the village-level.

In quantitative analysis, we first summarized background characteristics of youth interviewed for the mobile surveys. Additionally, responses from youth and household interviews were summarized by thematic area. In our main quantitative analyses, we longitudinally examined changes in youth outcomes relative to a pre-COVID period using multivariate adjusted regression models, among the same individuals. Changes in dichotomous outcomes of interest were examined using generalized linear models and we report risk ratios (RR) and 95% confidence intervals (CI). For continuous outcomes, we ran OLS regressions and we report estimated *β* coefficients with standard errors (SE). Regression models include as the independent variable a binary indicator of time (= 1 if 2020) to examine changes post-COVID. All regressions control for age, gender, and district x village size dummy variables. Standard errors were adjusted for clustering at the village-level. Analyses were conducted separately for households and youth surveys. All quantitative analyses were conducted using Stata Version 16.

For SMS interviews, we summarized responses over time among the panel of youth (households) who answered all SMS messages.

The qualitative interviews were double coded by three research team members, two of whom also conducted interviews. The coders used a codebook composed of codes drawn from the literature and the team’s previous work with this population, as well as emergent codes. All discrepancies were discussed by the coding team before beginning analysis. We used a thematic analysis approach to analyze the qualitative data (64, 65).

## Results

### Youth sample characteristics

There was a total of 760 youth included in this analysis from the Round 0 SMS data collection. The characteristics of this sample are shown in Table 1. 52% of the sample lived in Iringa and 48% in Mbeya. The average age of youth in this sample was 19.95 years. 58% of the entire sample were male, with 22% reporting being married. Table 1 also shows these characteristics stratified by sex. There are no statistically significant differences between the aforementioned characteristics when comparing males and females, with the exception of marriage. Significantly more females reported being married compared to males in this sample (26 vs. 19%, *p* = 0.014). These marriage proportions are comparable for females, but higher for males in the 15-24-year-old age group than in the 2015–2016 Tanzania DHS (27 vs. 9%, respectively) [37]. Additionally, differences between the eligible sample (*n* = 1,727) from which 1,066 adolescents were selected due to budgetary constraints, and ultimately 760 were interviewed (71% response rate), are shown in Supplementary Table 1. The eligible and attained sample are very similar, and characteristics for which we find statistically significant differences demonstrate small practical differences (e.g., there is a 1% point difference in region of residence; average age of 18.73 v. 18.84 years; educational attainment 7.93 years v. 8.16 years).

**Table 1:** Demographic characteristics, mental health, and coping mechanisms of youth, stratified by gender (*N* = 760)

	Percentage/Mean			
	Pooled	Females	Males	*p*-value
Region		N=344	N=416	
Iringa	52.24	51.45	52.88	0.694
Mbeya	47.76	48.55	47.12	0.694
Age (years)	19.95	19.93	19.97	0.741
Married	21.84	25.87	18.51	0.014
Mental Health				
Poor sleep past 7 days	16.84	14.83	18.51	0.177
Bothered by things - past 7 days	8.03	7.56	8.41	0.666
Started a sexual relationship with someone in order to get things you need, such as money or gifts - past 12 months (N = 343)	-	4.37	-	-
Engaged in negative coping strategy index (range 0–5)	1	0.93	1.06	0.102

### Household economic conditions and youth time use during COVID-19

Economic impacts attributed to the COVID-19 pandemic were widespread in the qualitative interviews, and most participants described them as major. The pandemic led to a series of problems that complicated economic activity, including participants’ fear of being in crowds, closures of some businesses, and limitations to transportation of goods and supplies using public transportation. Travel limitations (mostly self-imposed out of fear) were particularly challenging. Many of the interviewees’ households were involved in buying and selling produce and other items. As a male community member from Iringa explained, “People could no longer travel to other places to do business, for example going to markets to purchase goods. The effects of COVID-19 on the economy are evident in the disruption of the transportation of goods from where they are available to the consumer.” Another Iringa community member explained how supply chains had been disrupted in her area: “[A farmer] will grow his vegetables here in Mafinga, and a person from Iringa will not be able to come here to take the consignment of vegetables to go and sell the produce the farmer cultivated.” Several participants noted that produce rotted before they could sell it, even though many people were going hungry. The travel limitations also negatively impacted future food production, as importation of needed fertilizer was limited.

Fear was a commonly discussed barrier related to economic activity. Even when people had crops or other goods to sell, they “were afraid of going out to sell due to the pandemic,” according to a 21-year-old female participant in Mbeya. Few people came to the typically crowded markets to shop. As a 21-year-old male in Mbeya reported, “My mother goes to the market and returns [with] all items unsold.” Several interviewed participants said they had lost their jobs in shops, restaurants, and as guards during the pandemic. Casual work was difficult to pick up, as employers “were afraid people from other families might be sick and might infect them,” according to a female community member in Iringa.

At both the community and youth levels, qualitative interview participants reported improving economic conditions as the first wave of the COVID-19 pandemic abated in 2020, though the reopening threatened greater risk of COVID-19 spread. However, they were facing ongoing challenges, as they had spent their limited savings during the closures and subsequently many struggled to reopen their businesses without capital. For example, participants mentioned difficulties buying fertilizer and new seeds for farming and building materials for construction.

When schools closed, some children and youth spent more time doing household chores, such as fetching water or gathering firewood, or contributing to family economic activities. As a female community member in Iringa explained, “Some were even selling vegetables, without worrying about the presence of diseases, for the economy of the household.” Participants said that children engaged in activities such as collecting scrap metal, farming, gathering garbage, and selling mandazi, second-hand clothes, and produce. One 18-year-old participant in Iringa said of her siblings, “when I was alone at home, I had a lot of work to do, but during the time when they were at home, they really helped me a lot with work.” Hours spent on domestic chores in the past day among the entire sample (*N* = 760) were highest for caring for children, cooking, and cleaning (1.66 h) and collecting water or firewood (1.16 h) (Table 2). Females spent significantly more time on all domestic chores the day prior to the interview than males.

**Table 2:** Youth economic and time use outcomes, stratified by gender (*N* = 760)

	Mean/Percentage					
	Pooled	Females		Males		p-values
Time Use - yesterday (hours)						
Collecting water or firewood	1.16	1.49		0.88		< 0.001
Collecting nuts or other tree fruits, honey or other products from forest	0.24	0.33		0.18		0.009
Caring for children, cooking, cleaning	1.66	2.58		0.89		< 0.001
Caring for elderly or sick household members	0.6	0.89		0.36		< 0.001
Changes at market household usually shops at - compared to March 2020 (*N* = 756)						
Prices increased more than usual	51.06	55.23		47.57		0.036
Tried to purchase food or essential items that household purchases frequently but was unable to purchase due to limited supply or they were not available in the market - compared to March 2020	38.36	40.12		36.89		0.364
Standard of living compared to March 2020						
Getting worse	15.13	15.7		14.66		0.692
Getting better	29.08	32.85		25.96		0.037
Staying the same	55.79	51.45		59.38		0.029
Food insecurity						
Any household member was not able to eat the kinds of foods preferred because of lack of resources - sometimes or often (past month)	26.84	31.98		22.6		0.004
Any household member had to eat some foods they really did not want to eat because of a lack of resources to obtain other types of food - sometimes or often (past month)	25.53	32.56		19.71		< 0.001
Either unable to eat preferred foods or ate unwanted foods	18.52	23.66		14.46		< 0.001
Water insecurity						
There has not been as much water to drink as you would like for you or anyone in your household (often or sometimes) - past month	6.45	9.01		4.32		0.009
Anyone in household had to go without washing hands after dirty activities (e.g., defecating or changing diapers, cleaning animal dung) because of problems with water (often or sometimes) - past month	5.66	9.01		2.88		< 0.001

**Fig. 4:**
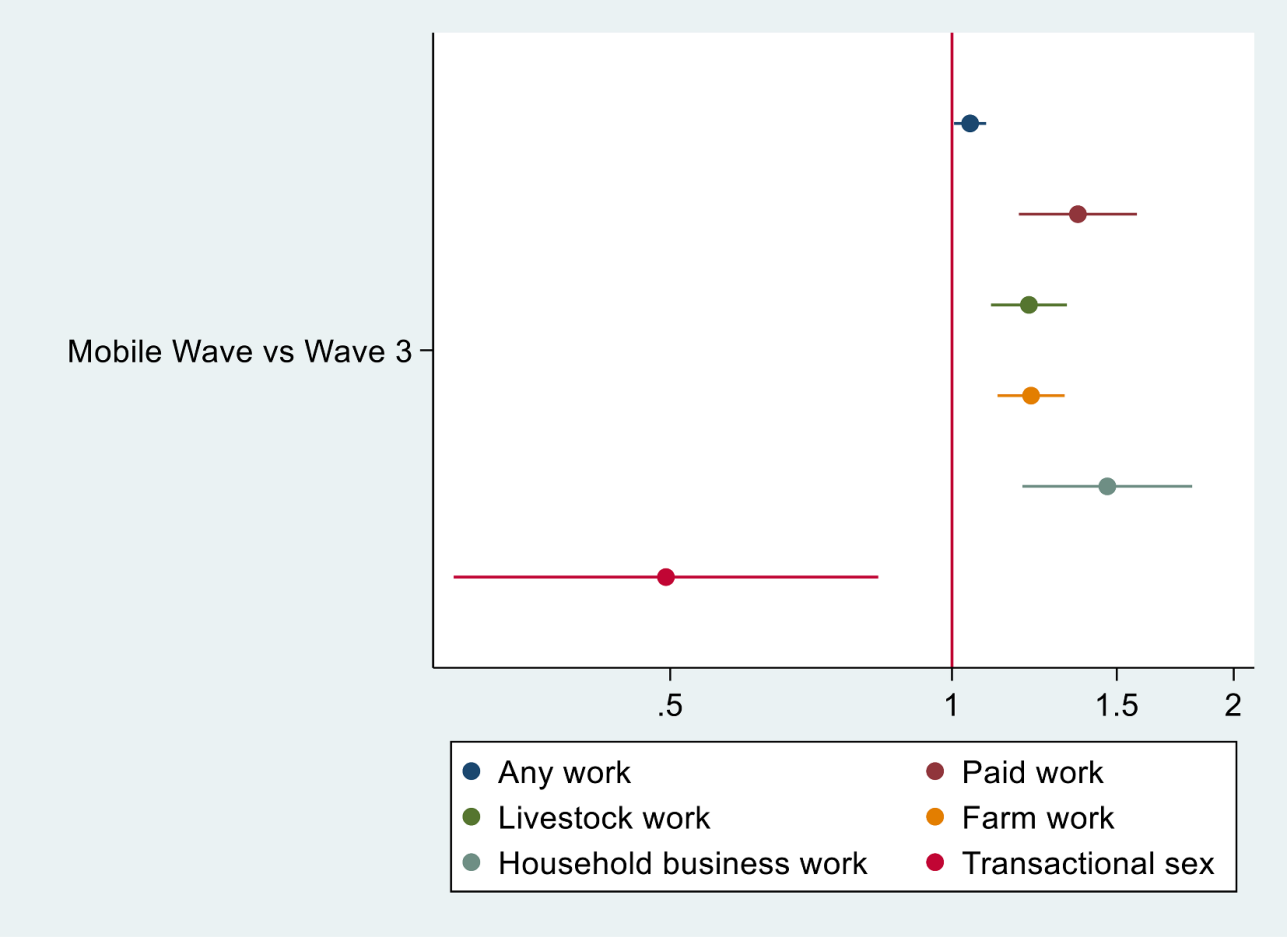
Risk ratios estimating the associated changes in economic activities and transactional sex over time (at the mobile wave versus wave 3) (*N* = 1520)

**Fig. 5:**
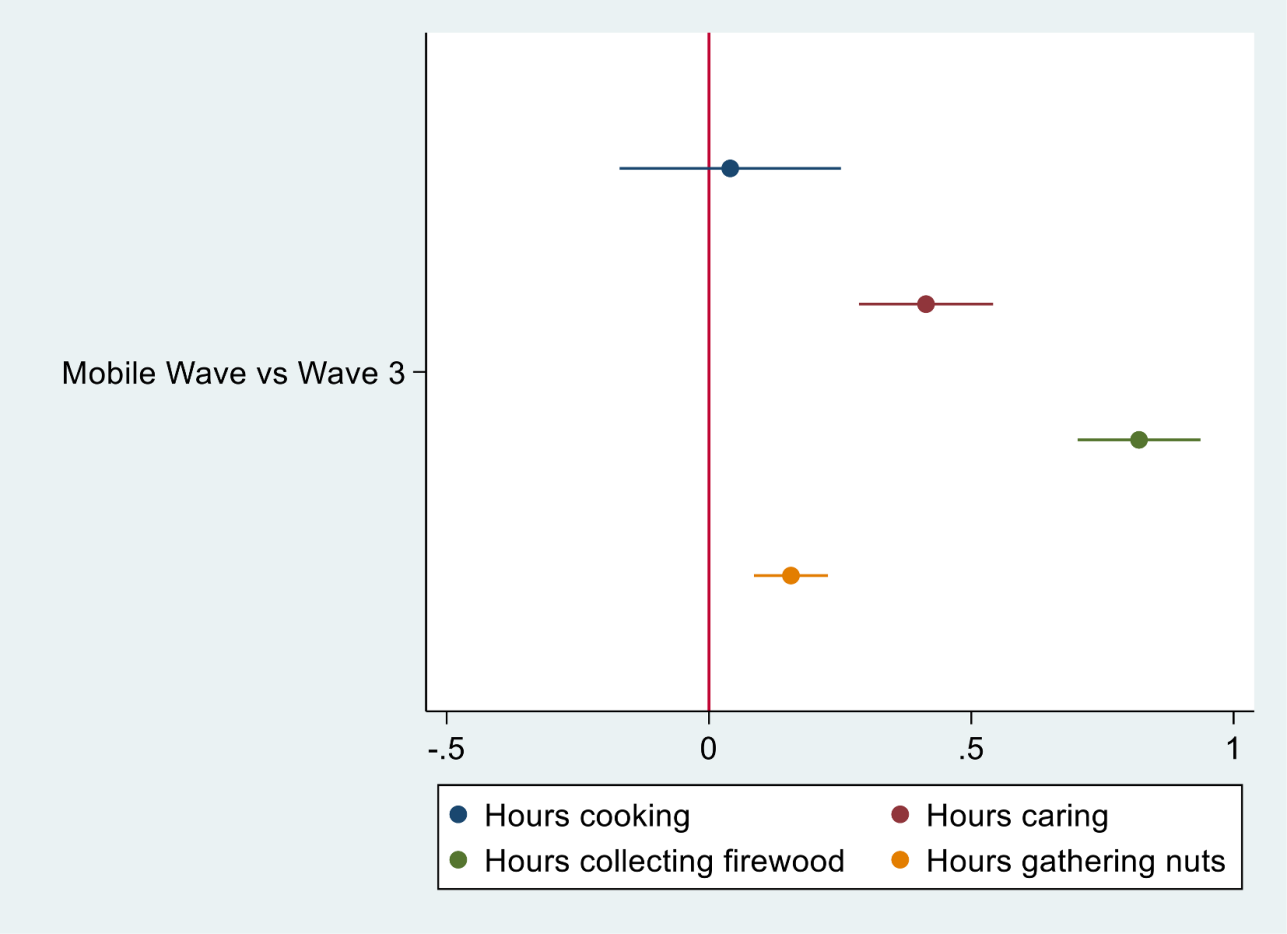
Estimates for hours spent on domestic chores at the mobile wave versus wave 3 (*N* = 1520)

Compared to the year prior (pre-COVID), in multivariate regressions controlling for age and other characteristics, youth were more likely to be engaged in the following activities: any work, farm work, livestock work, household business work, and paid work (Table 3). The risk ratios estimating the associated changes in economic activities and transactional sex over time are shown in Fig. 4. Participation in any work in the past week increased 5% from wave 3 to the mobile wave data collection wave. Farm work, livestock tending, household business, and paid work all increased 21, 21, 47, and 36% between waves, respectively. Examined separately by gender, we find that males, but not females, were more likely to be engaging in household business activities or paid work as compared to one year prior (Supplementary Tables 2 and 3). However, females, but not males, were more likely to be engaging in any work (including unpaid). Both males and females reported increases in farm work and livestock tending as compared to one year prior. Youth reported spending more hours caring for the elderly, gathering firewood, and collecting nuts or other tree fruits, honey, or other products from forests in the past day, as compared to one year prior (Table 3; Fig. 5). This is consistent with reports in the qualitative interviews. When examining total hours spent in activities by gender, we found males reported engaging in more hours of all chores, while females reported spending more hours caring for elderly and gathering firewood and nuts compared to pre-COVID (Supplementary Tables 4 and 5).

Heavy work for children enrolled in school seemed relatively rare during this period, however, many respondents reported that the members of their households were trying to stay home and social distance during the pandemic. “A large percentage remained locked up in their homes,” said one female community member in Iringa. Referring to their siblings, one 19-year-old in Iringa said, “Because we needed to protect them a lot they did not do any activities, they had to stay inside.” Additionally, they noted that since so many adults had lost their jobs, for example in the tourism or timber industries, there was great competition for any casual labor work that was available, and children were unlikely to be selected.

When asked about changes in their standard of living compared to six months ago (in quantitative surveys [Table 2]), 56% reported that it was staying the same, 15% reported it was getting worse, and 29% reported it was getting better. Females were more likely than males to report improvements in their standard of living. Households were asked similar questions on standard of living in the SMS surveys (Table 4 and Supplementary Table 4). Among the households interviewed at SMS round 0 (*N* = 542), 19%, 22%, and 59% of households reported worse, better, or unchanged standard of living since March 2020 (Table 4). Among the full panel sample of households interviewed at all SMS rounds (*N* = 95), reports of better standard of living increased to 39% at SMS round 1, levelled off at SMS round 2 and 3, and, ultimately decreased to 29% of households reported better standard of living at SMS round 4 (Supplementary Table 4). Reporting of worse standard of living ranged from 16% at Round 3 to 21% at Rounds 2 and 4 (Supplementary Table 4).

**Table 3:** Associations between mobile wave versus wave 3 and outcomes in time use and hours dedicated to chores

	Farm work	Livestock Work	Household business work	Paid work	Attends School	Tran-sactional sex, females only	Hours cooking	Hours caring for elderly	Hours gathering firewood	Hours gathering nuts
	RR	RR	RR	RR	RR	RR	β	β	β	β
Mobile Wave (ref = Wave 3)	1.21**	1.21**	1.47**	1.36**	1.11	0.50*	1.04	1.51**	2.27**	1.17**
	(1.12–1.32)	(1.10–1.33)	(1.19–1.81)	(1.18–1.58)	(0.92–1.33)	(0.25–1.00)	(0.84–1.29)	(1.33–1.72)	(2.02–2.55)	(1.09–1.25)
Age (years)	1	0.97	1.07*	1.06**	0.61**	1.03	1.03	0.98	1.01	1
	(0.96–1.03)	(0.93–1.00)	(1.00–1.13)	(1.02–1.10)	(0.53–0.70)	(0.84–1.26)	(0.97–1.09)	(0.94–1.01)	(0.98–1.05)	(0.98–1.03)
Female	0.98	0.99	0.77*	0.51**	1.29*		6.99**	1.37**	1.46**	1.06
	(0.89–1.08)	(0.91–1.07)	(0.61–0.97)	(0.43–0.61)	(1.01–1.66)		(5.55–8.81)	(1.19–1.57)	(1.32–1.60)	(0.99–1.15)
*N*	1,520	1,520	1,520	1,520	1,520	688	0.25	0.07	0.22	0.02
							1,520	1,519	1,520	1,520

** *p* < 0.05; *** *p* < 0.01 *Notes* regressions also control for district x size dummy variables used in sampling (coefficients not shown)

### Consequences of COVID-19-related economic stress on youth and their households

**Food insecurity and availability of goods.** The economic situations of the households at the Round 0 SMS survey are reported in Table 4. In the month prior to the interview, 39% of household respondents reported that they or a household member had to eat food they did not want due to lack of resources. Females were more likely than males to report food insecurity (Table 2). Over time, as reported in youth SMS surveys, we see an increasing percentage of youth in the panel sample who reported eating unwanted foods due to limited resources in October, November, and December (Supplementary Table 4).

**Table 4:** Household economic situation, Household SMS Surveys (*N* = 542)

	Percentage
Household member had to eat some foods they really did not want to eat because of a lack of resources - past month	39.48
Lack of money prevented household from sending children to school (households with children only) (*N* = 504)	15.28
Standard of living compared to March 2020	
Getting worse	18.45
Getting better	22.32
Staying the same	59.23
District	
Mufindi/Mafinga	53.32
Rungwe/Busokelo	46.68

Food prices and availability were also raised frequently in the qualitative interviews. Prices went up during the first wave of the COVID-19 pandemic, especially for sugar, beans, tomatoes, and other basic commodities. One Mbeya youth, a 20-year-old male, said, “When you went to the market you found prices were up while the money you had was not enough. As a result, you couldn’t buy anything.” Half of the respondents in the quantitative survey reported that prices had increased more than usual in the markets they usually shopped at, as compared to March 2020 (Table 2).

Participants also reported that some types of food were unavailable at any price during this period. A 20-year-old male participant in Mbeya said, “Getting supplies was a challenge, because they were not available when you wanted them.” Another 20-year-old male in Mbeya said he had “problems getting meat or fish.” In quantitative surveys, 38% of youth reported difficulties finding items (for example, sugar) in the market that their household typically buys (Table 2).

**Education.** Among households with children, 15% reported a lack of money as a barrier to sending children to school (Table 4). The percentage of households unable to send their children to school due to lack of money approximately doubled throughout subsequent interview rounds (Supplementary Table 4). This was not reported as a major issue in the qualitative interviews, however. A female community leader in Iringa explained, “There was also an effort to follow-up and make sure children returned to school. Therefore, there were not many cases of children not reporting to school…many reported because we took steps early.”

There were isolated cases of economic pressures leading to school enrollment issues reported in the interviews, however. One interviewed youth said that his sibling’s return to school after the COVID-19 closures was delayed due to the household’s financial constraints. Sending children to school incurs financial cost, even with free public school tuition, and COVID-19’s effects on the economy made this even more difficult than it was typically. For example, costs include those of shoes, uniforms, books, or in the case of boarding schools, the cost of lodging and food (as many secondary schools are located far from children’s homes and boarding is necessary). A 20-year-old male in Mbeya discussed the challenges he had in getting one of his siblings back to school. “He did not return immediately because he didn’t have some of the requirements. How could he go to school without them? He had to wait until we got them… He returned to school after two weeks… Since there is no one to run to for help all I could do was to tell him to be patient, and God is great, I got the money and he returned to school.”

**Youth mental health.** Table 1 presents the findings for mental health. Nearly 17% of the sample (*N* = 760)—15% of females and 19% of males —reported sleeping well rarely or sometimes in the past seven days. Fewer youth (8%) of youth reported being unusually bothered by things most or all the time in the past seven days. These percentages were similar across males and females (Table 1).

During the COVID-19 pandemic, interview participants reported experiencing heightened fear and anxiety not only about their health, but also about their ability to provide for themselves and their families. In qualitative interviews, fear was expressed by 25 of 44 youth participants and 15 of 16 community participants. One participant, a 20-year-old male in Mbeya, said, “When this disease started we were spending the day at home because we were afraid. We would watch the news, and it would be announced so many died here, a hundred there and a thousand in another place.” Fear led participants to change their productive activities to protect themselves. In Iringa, a 19-year-old male reported, “Truly, difficulties were there because you know in the market there are crowds of people, so much that sometimes we miss something you need. You are forced to stay [home] because you are scared.” A male community leader in Iringa said, “there was fear that spread all over the community.”

Anxiety among household members was mentioned by 21 of 44 youth participants in qualitative interviews. A 19-year-old male participant in Iringa explained, “Because you get surprised when you have a cough, you find yourself starting to worry saying ‘has this disease got me or what’ you start being anxious since you have suddenly started coughing, you say ‘or this disease has caught up with me’ Truly, a lot of anxiousness was there.” Several participants, including a 20-year-old woman in Mbeya, said they were anxious about people in the community not following COVID-19 prevention protocols. “For example you would go into a shop; you find there is no water for washing hands… it just caused us anxiety.” The availability of food in local markets, the risk of going out to obtain it, and the household’s ability to pay for it were all sources of anxiety.

**Negative coping strategies.** A number of coping strategies were used by participants’ households to meet the financial challenges brought on by the COVID-19 pandemic. Seven of the 44 youth participants in the qualitative interviews reported selling livestock—chickens, pigs, and cows—or land. In four cases, participants said that they or someone else in their households had taken loans. In a few cases, participants changed to different businesses or became subsistence farmers, to ensure that the household would at least have food. There were barriers to changing economic activities, however. As a male community member in Iringa explained, “This created some difficulties, as you need to get used to the new activity that you are forced to do… This might require capital that you might not have.”

Among females in the quantitative survey (*N* = 343), 4% reported starting a sexual relationship in order to obtain money or gifts in the past 12 months (Table 1). Youth also reported that, on average, their household engaged in one negative coping strategy in response to household shocks (Table 1). This reported value was similar among males and females. Three participants in the qualitative interviews reported transactional sexual relationships, though one of these relationships pre-existed COVID-19. Risk of engaging in transactional sex (among females only) was 50% lower at the mobile wave compared to wave 3 (Table 3).

## Discussion

This study leverages a longitudinal sample from the ongoing Ujana Salama pilot impact evaluation in order to summarize the impacts of the COVID-19 pandemic on economic security, time use activities, coping strategies, metal health, and sexual exploitation among a sample of youth from the Southern Highlands of Tanzania. This innovative study collected quantitative and qualitative data using mobile phone surveys administered monthly to youth and household heads where youth live. We find numerous and salient impacts of the COVID-19 pandemic on the economic well-being and health of households and youth. We also find there to be gendered responses to the impacts of the COVID-19 pandemic among these youth.

We observed major impacts of the pandemic on economic security as prices of goods were reported to increase while market availability of goods was reported to decrease relative to March 2020. Females reported more hours engaging in caregiving, cooking, and collecting water, firewood and nuts compared to males. On average, youth engaged in one negative coping mechanism in response to the economic impacts of the COVID-19 pandemic. Both males and females experienced increases in time use activities generally and hours dedicated to chores during the mobile wave compared to wave 3. Food and water insecurity were prevalent among these youth and households, more so among females than males. Further, reports of food insecurity by household heads and youth increased over time as the COVID-19 pandemic persisted.

Though only 14% of this sample of youth were attending school pre-COVID, all returned to schools when they reopened. Qualitative interviews described this to be a result of community support and work to ensure children attended school upon reopening and likely also due to the fact that schools had only closed for two months. This is considerably shorter than the duration of school closures in neighboring countries such as Uganda and may have contributed to fewer impacts on school dropout, but also indirectly on adolescent pregnancy and other outcomes. Despite some households reporting being unable to send their children back to school because of lack of finances, youth school attendance increased over time. Increased pregnancies among school-aged girls earlier in the pandemic were reported in qualitative interviews. However, reported transactional sex decreased among females in the mobile wave compared to wave 3.

The findings of our study are comparable to those of studies with similar research objectives for certain domains. For example, Jones, Guglielmi [38] and colleagues present qualitative data to suggest that many adolescents in their urban Ethiopia sample returned to school in the final quarter of 2020, while the other three focal countries (Jordan, Bangladesh, and Palestine) did not see such returns to school among those adolescent samples. In Kenya, 84% of girls and 92% of boys were re-enrolled in school by January 2021 [12]. Similarly, our study found that community efforts to return youth to school were successful and all of the 14% of the sample who were attending school at the time of COVID-related school closures returned. Gendered impacts of the pandemic were also observed as time dedicated to paid work and to unpaid domestic and care work increased for males and females, respectively [13, 38]. Our results suggest that females and males experienced increases in time use related to care giving and collection of firewood and nuts while males also increased time dedicated to paid work. Food insecurity measures were also observed to be higher among females compared to males among a Kenyan sample of adolescents, though the prevalence of food insecurity was much higher in Kenya (~ 75%) probably due to stricter COVID-19 restrictions compared to Tanzania [12].

Our study found that vulnerability of females as measured by transactional sex engagement in the past 12 months decreased (50% reduction), but these findings are in contrast to vulnerability-related findings in neighboring countries. For example, in rural Ethiopia, adolescent girls reported feeling greater pressure to be married during COVID [38]. Female vulnerability was also observed among an adolescent sample of females in Kenya, where 4% of 15–19 year old girls were pregnant or reported recently having a baby and 29% of adolescent girls reported getting married after the COVID-19 pandemic, 42% of whom got married after getting pregnant [12]. This suggests that in the Kenyan context, effects of the pandemic were accelerating adolescent pregnancy and in turn, adolescent marriage. Our study also found no significant differences in reliance on negative coping mechanisms in response to the changes and restrictions imposed due to COVID-19 by gender. This may in part be due to the fact that social distancing and prevention measures were limited and of short duration in Tanzania.

There are some limitations to this study that warrant discussion. Given the mobile data collection interview time constraints, a limited number of questions were asked and individual items from full scales were used as opposed to full scales often asked in face-to-face settings. For example, the use of two CES-D10 items limited our ability to measure impacts on a multidimensional measure of depression. However, somatic symptoms (e.g. troubled sleep, bothered), as opposed to mood related symptoms (e.g. loneliness, feeling depressed), have been identified as more salient expressions of ill-mental health among Tanzanian populations [39]. Additionally, due to the sensitive nature of transactional sex questions, our results may be underestimates. Social distancing, school closures, and household economic impacts of the COVID-19 pandemic may have adverse impacts on youth mental health that might take longer to materialize. Nevertheless, the duration of closures and other preventive measures was short-lived in Tanzania and thus may have few impacts. Another limitation is that these findings are not generalizable to all youth in Tanzania. The sample is primarily rural and drawn from households participating in a national social protection program. Thus, they are among the poorest and most vulnerable households in Tanzania. This population is vulnerable to health impacts of macroeconomic conditions, and their health and ability to access services may be adversely affected through more economic channels. Finally, we cannot disentangle the impacts of COVID-19 from those of the PSSN payment delays (March 2019 to September 2020), which overlapped. Both of these created economic hardship for the households studied.

Based on our findings, we recommend efforts to expand social protection during the pandemic and other macroeconomic shocks. Some households who did not meet eligibility criteria during previous program roll-out periods may subsequently have been impoverished due to the recent crisis, and thus additional targeting and horizontal program expansion should be considered. Other forms of economic support are also needed. The most common source of anxiety and worry in this study was economic-related, and so economic strengthening initiatives can mitigate these adverse impacts. Initiatives may include scaling up agricultural extension programming to promote resiliency and increased productivity of crop production and livestock keeping. Other initiatives might include grants to support small businesses. These, in turn, can help mitigate negative coping strategies such as selling of agricultural assets and livestock, or forcing children to drop out of school. Finally, we recommend scaling up community-based mental health programming. In this setting, with limited capacity in terms of professionally trained psychologists, psychiatrists, clinical social workers, and other mental health professionals, community-based interventions are a promising strategy to respond to mental health needs, which are exacerbated by poverty.

## Conclusion

This mixed-method mobile study examined impacts of COVID-19 on youth well-being in Tanzania. Economic impacts were the most salient, and to cope, youth and their households engaged in several negative coping mechanisms included changing eating patterns, taking on loans, and selling livestock or land. These study findings highlight several vulnerabilities faced by youth and their families during the pandemic underscore the potential opportunity to leverage this moment in the wake of COVID-19 to promote policies and programs which address risks youth face.

### Electronic supplementary material

Below is the link to the electronic supplementary material.

Supplementary Material 1: Mean Characteristics for Mobile Survey Eligible Youth Sample

Supplementary Material 2: Descriptive Summary of Outcomes for the Full Panel Sample of Households, by Round

Supplementary Material 3: Timing of interviews and samples

Supplementary Material 4: Youth outcomes, full panel of SMS surveys, by round

Supplementary Material 5: Associations between Mobile Wave versus Wave 3 and outcomes in time use and hours dedicated to chores among males

Supplementary Material 6: Associations between Mobile Wave versus Wave 3 and outcomes in time use and hours dedicated to chores among females

Supplementary Material 7: Study Design and Sampling Information

Supplementary Material 8: Youth Interview Protocol

## Data Availability

Data used in this study are not publicly available. Requests to access must be approved by UNICEF Tanzania, the Government of the Republic of Tanzania, and the University at Buffalo. Requests should be made in writing to tiapaler@buffalo.edu.

## Abbreviations

CES-D10: 10-item Centers for Epidemiological Studies-Depression Scale
CI: Confidence interval
COVID-19: Coronavirus pandemic of 2019
DC: District Council
HWISE: Household Water Insecurity Experiences
MoHCDGEC: Ministry of Health, Community Development, Gender, Elderly and Children
OLS: Ordinary least squares
PSSN: Productive Social Safety Net
RR: Risk ratio
SARS-CoV-2: Severe Acute Respiratory Syndrome Coronavirus 2
SE: Standard error
SMS: Short message service
SOP: Standard Operating Procedures
STAG-IH: Strategic Technical Advisory Group for Infectious Hazards
TACAIDS: Tanzania Commission for AIDS
TASAF: Tanzania Social Action Fund
TC: Town Council
UNICEF: United Nations Children's Fund
USD: United States Dollar
WHO: World Health Organization
