# Supplementary material for: Economic vulnerabilities, mental health, and coping strategies among Tanzanian youth during COVID-19
Source: BMC Public Health. 2024 Feb 22;24:577. doi: 10.1186/s12889-024-18074-z (PMC10885560; doi:10.1186/s12889-024-18074-z)
Supplement: Supplementary file 1 — Supplementary Material 1: Mean Characteristics for Mobile Survey Eligible Youth Sample [file 12889_2024_18074_MOESM1_ESM.docx]

**Supplementary table 1. Mean Characteristics for Mobile Survey Eligible Youth Sample**

|  | Full Cohort in 2019 | Eligible Sample | p-value  (Full cohort v. eligible)* | Mobile Sample | p-value  (eligible v. mobile)* |
| --- | --- | --- | --- | --- | --- |
| Iringa | 0.51 | 0.51 | 0.976 | 0.52 | **<0.001** |
| Mbeya | 0.49 | 0.49 | 0.998 | 0.48 | **<0.001** |
| Age | 18.11 | 18.73 | **<0.001** | 18.84 | **0.013** |
| Attends School | 0.33 | 0.23 | **<0.001** | 0.21 | 0.105 |
| Highest Grade Completed | 7.85 | 7.93 | **<0.001** | 8.16 | **0.003** |
| Any Economic Activities | 0.81 | 0.83 | **0.001** | 0.84 | 0.313 |
| Farm work | 0.54 | 0.55 | 0.356 | 0.54 | 0.539 |
| Livestock work | 0.51 | 0.50 | 0.361 | 0.50 | 0.703 |
| Fishing for household | 0.03 | 0.02 | 0.184 | 0.03 | 0.796 |
| Household business | 0.16 | 0.17 | **<0.001** | 0.19 | 0.139 |
| Paid work | 0.26 | 0.29 | **<0.001** | 0.31 | 0.297 |
| Any chores | 0.88 | 0.88 | 0.321 | 0.89 | 0.447 |
| Collecting water | 0.71 | 0.70 | **0.026** | 0.70 | 0.743 |
| Collecting firewood | 0.29 | 0.29 | 0.732 | 0.27 | 0.102 |
| Gathering nuts | 0.07 | 0.07 | 0.748 | 0.05 | **0.030** |
| Taking care of children | 0.70 | 0.70 | 0.725 | 0.71 | 0.485 |
| Taking care of elderly or sick | 0.17 | 0.17 | 0.489 | 0.16 | 0.311 |
| Depressed (CESD=>10) | 0.23 | 0.24 | **0.013** | 0.24 | 0.777 |
| Well-being Stress | 2.75 | 2.90 | **<0.001** | 2.77 | 0.128 |
| Risk-related Stress | 0.37 | 0.37 | 0.638 | 0.33 | 0.161 |
| Relationship Stress | 0.42 | 0.45 | **0.031** | 0.37 | **0.028** |
| Quality of Life | 4.82 | 4.75 | **0.010** | 4.73 | 0.759 |
| Tested for HIV (past year) | 0.46 | 0.50 | **<0.001** | 0.50 | 0.829 |
| Sought SRH services | 0.26 | 0.30 | **<0.001** | 0.31 | 0.480 |
| Single | 0.92 | 0.90 | **<0.001** | 0.91 | 0.684 |
| Married | 0.07 | 0.09 | **<0.001** | 0.09 | 0.928 |
| Separated | 0.01 | 0.01 | **<0.001** | 0.01 | 0.196 |
| *N* | 2,191 | 1,727 |  | 760 |  |
| Looking for job | 0.14 | 0.17 | **<0.001** | 0.18 | 0.675 |
| *N* | 1,096 | 773 |  | 338 |  |
| Locus of Control | 3.29 | 3.31 | **<0.001** | 3.31 | 0.983 |
| Self-Esteem | 3.82 | 3.80 | 0.194 | 3.78 | 0.338 |
| Social Support | 3.92 | 3.92 | 0.827 | 3.91 | 0.810 |
| *N* | 2,190 | 1,726 |  | 760 |  |
| Ever pregnant | 0.30 | 0.40 | **<0.001** | 0.37 | 0.178 |
| *N* | 1,001 | 753 |  | 344 |  |
| Ever impregnated a female | 0.04 | 0.05 | **<0.001** | 0.04 | 0.645 |
| *N* | 1,190 | 974 |  | 416 |  |
| Emotional Violence | 0.09 | 0.10 | 0.105 | 0.09 | 0.353 |
| Physical Violence | 0.11 | 0.09 | **0.004** | 0.08 | 0.178 |
| Any Violence | 0.18 | 0.16 | 0.138 | 0.14 | 0.127 |
| *N* | 1,033 | 805 |  | 336 |  |

*p-values determined by regressions comparing wave 3 to eligible sample and eligible sample to mobile sample while controlling for district and size dummy variables and clustering standard errors at the village level.

Farming, livestock, fishing, family business, paid work, and job hunting refer to the past 7 days

Any chores, collecting water, firewood, nut, and providing care (children or elderly) refer the past 24 hours

Experiences of violence (emotional, physical) are in reference to the past year
