# Supplementary material for: Economic vulnerabilities, mental health, and coping strategies among Tanzanian youth during COVID-19
Source: BMC Public Health. 2024 Feb 22;24:577. doi: 10.1186/s12889-024-18074-z (PMC10885560; doi:10.1186/s12889-024-18074-z)
Supplement: Supplementary file 2 — Supplementary Material 2: Descriptive Summary of Outcomes for the Full Panel Sample of Households, by Round [file 12889_2024_18074_MOESM2_ESM.docx]

| **Supplementary table 4. Descriptive Summary of Outcomes for the Full Panel Sample of Households, by Round** | | | | | |
| --- | --- | --- | --- | --- | --- |
|  | Round 0 | Round 1 | Round 2 | Round 3 | Round 4 |
| District |  |  |  |  |  |
| Mufindi/Mafinga | 0.47 | 0.47 | 0.46 | 0.47 | 0.47 |
| Rungwe/Busokelo | 0.53 | 0.53 | 0.51 | 0.53 | 0.53 |
| Received TASAF payment | 0.05 | 0.59 | - | - | 0.24 |
| Household member ate unwanted foods | 0.47 | 0.62 | 0.61 | 0.56 | 0.6 |
| Lack of money prevented household from sending children to school | 0.12 | 0.26 | 0.33 | 0.29 | 0.25 |
| Lack of money prevented household from sending children to school (households with children only) | 0.12 (N=90) | 0.29 (N=87) | 0.37 (N=84) | 0.35 (N=79) | 0.29 (N=82) |
| Standard of living |  |  |  |  |  |
| Better | 0.23 | 0.39 | 0.33 | 0.33 | 0.29 |
| Worse | 0.19 | 0.18 | 0.21 | 0.16 | 0.21 |
| Same | 0.58 | 0.43 | 0.46 | 0.52 | 0.49 |
| N | 95 | 95 | 95 | 95 | 95 |
