# Supplementary material for: Economic vulnerabilities, mental health, and coping strategies among Tanzanian youth during COVID-19
Source: BMC Public Health. 2024 Feb 22;24:577. doi: 10.1186/s12889-024-18074-z (PMC10885560; doi:10.1186/s12889-024-18074-z)
Supplement: Supplementary file 3 — Supplementary Material 3: Timing of interviews and samples [file 12889_2024_18074_MOESM3_ESM.docx]

**Supplementary figure 1. Timing of interviews and samples**
