# Supplementary material for: Economic vulnerabilities, mental health, and coping strategies among Tanzanian youth during COVID-19
Source: BMC Public Health. 2024 Feb 22;24:577. doi: 10.1186/s12889-024-18074-z (PMC10885560; doi:10.1186/s12889-024-18074-z)
Supplement: Supplementary file 4 — Supplementary Material 4: Youth outcomes, full panel of SMS surveys, by round [file 12889_2024_18074_MOESM4_ESM.docx]

| **Supplementary table 5. Youth outcomes, full panel of SMS surveys, by round** | | | | |
| --- | --- | --- | --- | --- |
|  | Round 1 | Round 2 | Round 3 | Round 4 |
| District |  |  |  |  |
| Mufindi/Mafinga | 0.53 | 0.53 | 0.53 | 0.53 |
| Rungwe/Busokelo | 0.47 | 0.47 | 0.47 | 0.47 |
| Attends School | 0.25 | 0.28 | 0.29 | 0.29 |
| Hours spent in farming, tending livestock, or fishing |  |  |  |  |
| Increased | 0.4 | - | - | 0.4 |
| Decreased | 0.19 | - | - | 0.25 |
| Same | 0.41 | - | - | 0.35 |
| Ate unwanted foods due to limited resources | 0.51 | - | 0.57 | 0.58 |
| Felt bothered by things that don't typically bother | 0.11 | - | - | - |
| N | 169 | 169 | 169 | 169 |
