# Supplementary material for: Economic vulnerabilities, mental health, and coping strategies among Tanzanian youth during COVID-19
Source: BMC Public Health. 2024 Feb 22;24:577. doi: 10.1186/s12889-024-18074-z (PMC10885560; doi:10.1186/s12889-024-18074-z)
Supplement: Supplementary file 5 — Supplementary Material 5: Associations between Mobile Wave versus Wave 3 and outcomes in time use and hours dedicated to chores among males [file 12889_2024_18074_MOESM5_ESM.docx]

**Supplementary table 3. Associations between Mobile Wave versus Wave 3 and outcomes in time use^a^ and hours dedicated to chores^b^ among males**

|  | **Any work** | **Farm work** | **Livestock Work** | **Household business work** | **Paid work** | **Attends School** | **Hours cooking** | **Hours caring for elderly** | **Hours gathering firewood** | **Hours gathering nuts** |
| --- | --- | --- | --- | --- | --- | --- | --- | --- | --- | --- |
| Mobile Wave vs Wave 3 | 1.02 | 1.18** | 1.19** | 1.64** | 1.46** | 1.03 | 0.37** | 0.21** | 0.65** | 0.10* |
|  | (0.98 - 1.06) | (1.06 - 1.32) | (1.06 - 1.35) | (1.25 - 2.15) | (1.26 - 1.68) | (0.78 - 1.37) | (0.08) | (0.06) | (0.06) | (0.05) |
| Age (years) | 1.02 | 0.95 | 0.90** | 1.02 | 1.06* | 0.63** | -0.06* | -0.01 | -0.03 | -0.02 |
| District (ref: Iringa-small) | (1.00 - 1.03) | (0.91 - 1.00) | (0.86 - 0.95) | (0.95 - 1.11) | (1.01 - 1.11) | (0.52 - 0.75) | (0.02) | (0.02) | (0.02) | (0.01) |
| Iringa - large | 1.06 | 1.08 | 1.04 | 1.50 | 1.10 | 0.71 | 0.10 | 0.10 | 0.01 | -0.11 |
|  | (0.98 - 1.16) | (0.88 - 1.32) | (0.86 - 1.26) | (0.99 - 2.27) | (0.88 - 1.38) | (0.45 - 1.12) | (0.12) | (0.10) | (0.09) | (0.09) |
| Mbeya - small | 1.04 | 0.95 | 1.13 | 1.90* | 1.15 | 0.59 | -0.40** | 0.06 | -0.10 | -0.15 |
|  | (0.95 - 1.14) | (0.76 - 1.20) | (0.90 - 1.43) | (1.10 - 3.28) | (0.90 - 1.47) | (0.31 - 1.10) | (0.11) | (0.07) | (0.09) | (0.09) |
| Mbeya - large | 1.04 | 1.13 | 1.19 | 1.73** | 1.23 | 0.68 | -0.25* | 0.09 | -0.09 | -0.15 |
|  | (0.96 - 1.13) | (0.91 - 1.39) | (0.99 - 1.42) | (1.14 - 2.63) | (0.97 - 1.55) | (0.39 - 1.19) | (0.10) | (0.08) | (0.08) | (0.08) |
| *N* | 832 | 832 | 832 | 832 | 832 | 832 | 832 | 832 | 832 | 832 |

* *p*<0.10; ** *p*<0.05; *** *p*<0.01. **^a^**Within the past week. **^b^**Yesterday.
