# Supplementary material for: Economic vulnerabilities, mental health, and coping strategies among Tanzanian youth during COVID-19
Source: BMC Public Health. 2024 Feb 22;24:577. doi: 10.1186/s12889-024-18074-z (PMC10885560; doi:10.1186/s12889-024-18074-z)
Supplement: Supplementary file 6 — Supplementary Material 6: Associations between Mobile Wave versus Wave 3 and outcomes in time use and hours dedicated to chores among females [file 12889_2024_18074_MOESM6_ESM.docx]

**Supplementary table 2. Associations between Mobile Wave versus Wave 3 and outcomes in time use^a^ and hours dedicated to chores,^b^  among females**

|  | **Any work** | **Farm work** | **Livestock Work** | **Household business work** | **Paid work** | **Attends School** | **Transactional sex - females only** | **Hours cooking** | **Hours caring for elderly** | **Hours gathering firewood** | **Hours gathering nuts** |
| --- | --- | --- | --- | --- | --- | --- | --- | --- | --- | --- | --- |
| Mobile Wave vs Wave 3 | 1.08* | 1.27** | 1.25** | 1.24 | 1.17 | 1.20 | 0.50* | -0.38 | 0.65** | 1.02** | 0.22** |
|  | (1.00 - 1.17) | (1.11 - 1.46) | (1.08 - 1.45) | (0.93 - 1.65) | (0.85 - 1.60) | (0.97 - 1.48) | (0.25 - 1.00) | (0.22) | (0.12) | (0.09) | (0.05) |
| Age (years) | 1.03** | 1.03 | 1.02 | 1.13** | 1.06 | 0.58** | 1.03 | 0.15** | -0.03 | 0.06* | 0.03 |
| District (ref: Iringa – small) | (1.01 - 1.05) | (0.99 - 1.07) | (0.98 - 1.07) | (1.04 - 1.23) | (0.99 - 1.14) | (0.50 - 0.69) | (0.84 - 1.26) | (0.05) | (0.03) | (0.03) | (0.02) |
| Iringa - large | 0.95 | 0.88 | 0.71** | 0.59* | 0.96 | 1.43 | 1.51 | 0.16 | -0.17 | -0.19 | -0.14 |
|  | (0.87 - 1.04) | (0.74 - 1.06) | (0.59 - 0.87) | (0.37 - 0.94) | (0.62 - 1.49) | (0.75 - 2.73) | (0.52 - 4.42) | (0.23) | (0.14) | (0.11) | (0.07) |
| Mbeya - small | 0.97 | 0.78 | 0.82 | 1.58* | 0.61* | 1.82 | 2.37 | 0.71* | 0.21 | -0.05 | -0.05 |
|  | (0.87 - 1.09) | (0.56 - 1.08) | (0.66 - 1.03) | (1.02 - 2.46) | (0.38 - 0.97) | (0.93 - 3.53) | (0.73 - 7.75) | (0.30) | (0.19) | (0.13) | (0.08) |
| Mbeya - large | 0.95 | 0.84 | 0.76** | 1.31 | 0.94 | 1.28 | 1.75 | 0.90** | 0.27 | 0.18 | 0.11 |
|  | (0.86 - 1.06) | (0.69 - 1.03) | (0.63 - 0.93) | (0.83 - 2.08) | (0.60 - 1.49) | (0.66 - 2.48) | (0.62 - 4.91) | (0.27) | (0.17) | (0.12) | (0.10) |
| *N* | 688 | 688 | 688 | 688 | 688 | 688 | 688 | 688 | 687 | 688 | 688 |
