# Supplementary material for: Economic vulnerabilities, mental health, and coping strategies among Tanzanian youth during COVID-19
Source: BMC Public Health. 2024 Feb 22;24:577. doi: 10.1186/s12889-024-18074-z (PMC10885560; doi:10.1186/s12889-024-18074-z)
Supplement: Supplementary file 7 — Supplementary Material 7: Study Design and Sampling Information [file 12889_2024_18074_MOESM7_ESM.docx]

**Supplementary information 1. Study Design and Sampling Information**

The Ujana Salama impact evaluation comprises a cluster randomized control trial (cRCT) design and aims to examine impacts of an adolescent focused ‘Cash Plus’ intervention layered on top of Tanzania’s flagship social protection program, the Productive Social Safety Net (PSSN), implemented by the Tanzania Social Action Fund (TASAF). The Cash Plus intervention targeted to adolescents is layered onto the PSSN and comprises 1) livelihoods and life skills training, 2) mentoring and a productive grant, and 3) linkages to strengthened adolescent-friendly health services (UNICEF, 2017).

In this cRCT design, 130 clusters (communities) from four districts were randomized into control and treatment arms. Randomization was stratified by district and village size (large v. small villages) during a public randomization event with district-level government officials. In each district, village names were written on a piece of paper and placed into one of two hats (large villages v. small villages). District leaders then chose names from the hat and read them aloud, while the study coordinator wrote the village names down in order until all villages had been withdrawn. The top (first) half of the list was assigned heads and the second half tails. District officials completed a coin toss for each list (small and large villages) to assign villages to treatment.

All adolescents within the eligibility age range living in TASAF households in communities sampled were targeted for interviews, and sample size was determined based on power calculations of key outcomes from the study’s theory of change. Randomization took place in July 2017, after implementation of the baseline surveys (April – June 2017). The evaluation was a multi-year, longitudinal, mixed-method study comprised of baseline (2017), round 2 (2018), round 3 (2019), mobile surveys (2020), and round 4 (2021).
