# Supplementary material for: Economic vulnerabilities, mental health, and coping strategies among Tanzanian youth during COVID-19
Source: BMC Public Health. 2024 Feb 22;24:577. doi: 10.1186/s12889-024-18074-z (PMC10885560; doi:10.1186/s12889-024-18074-z)
Supplement: Supplementary file 8 — Supplementary Material 8: Youth Interview Protocol [file 12889_2024_18074_MOESM8_ESM.docx]

**Supplementary File. Youth Interview Protocol**

1. **Background**

A1. Please tell me about the people who live here in your household.

*Probe: Who lives with you, and how are they related to you?*

1. **Awareness of COVID-19**

B1. What do you know about COVID-19?

B2. What can people do to protect themselves from COVID-19?

B3. What measures has your household taken, if any, to protect yourselves?

*Examples, if needed: staying home more/entirely, avoiding touching people, wearing masks, using hand sanitizer or soap, not working outside the home, etc.*

B4. Where have you gotten information about COVID-19?

*Probe: Have you received information from social media, health workers, or prominent politicians?*

1. **COVID-19 and household economic situation**

C1. Please tell me about your household’s economic situation before and after the COVID-19 pandemic came to Tanzania.

C2. What productive activities did you engage in before? Have those activities changed now? If so, please explain.

C3. Have the productive activities of other household members changed due to COVID-19?

*Probe specifically regarding the work of any children in the household.*

C4. How have children who are out of school due to COVID-19 been participating in more economic activities for the household?

*Probe: Have any started working for pay or increasing number of hours worked? Are they contributing more to farming, livestock tending, or work in the household business?*

*Probe: How have these changes in children’s activities differed between girls and boys, generally?*

C5. Have you had difficulties getting any needed supplies at the market? Please explain, if so.

*Probe: Are your difficulties obtaining these items related to your household’s reduced income, increased prices in the market, or low supply of items in the market?*

C6. What are the greatest challenges your household is currently facing, with COVID-19?

C7. What support has your household received and what actions has your household taken to cope with these challenges?

*Probe: Have you received TASAF PSSN cash transfer, have they taken out loans or sold assets, have they increased casual labor engagement?*

C8. What else is your family doing to try to address these issues?

*Probe regarding reverse migration from urban areas to rural areas, if it does not come up on its own.*

C9. In the past 4 months, have you started a sexual relationship with someone to get things you need, such as food, basic necessities, or other items?

*Probe: What items did you need, and did your partner provide them for you? How long did this relationship last? Is it on-going?*

1. **COVID-19 and education**

D1. Were you enrolled in school before COVID-19?

*If so*: Do you intend to return to school when they reopen? Are there any factors that may prevent you from continuing with your schooling?

D2. Are there any younger children in this household who were enrolled in school before COVID-19?

*If so*: Will they return to school when they reopen? Are there any factors that may prevent them from continuing their schooling?

D3. *If anyone in the household was enrolled in school*: Please tell us what the students have been doing at home to continue learning, if anything.

Probe: *If students are continuing to learn at home*: Is anyone helping them learn at home? Who is primarily responsible for these children when they are at home?

D4. How do students feel about being home for this time?

1. **COVID-19 and health**

E1. Have you needed to seek medical care during COVID-19, either for routine matters such as check-ups, family planning, prenatal services, etc. or other serious matters such as delivery of a newborn or illness?

E2. *If so:* Did you have any trouble getting medical care?

*Probe: financial constraints, closures of medical providers, fear of COVID-19 contagion, family member prohibited you from seeking care, etc.*

E3. Is your household enrolled in the Community Health Fund? If so, in what way has this affected your household’s ability to seek health care?

E4. During the COVID-19 pandemic, have you felt any changes in your mental state?

E5. Have other members of your household had problems with stress and anxiety during this period?

E6. Have you ever felt unsafe in your home or community during COVID-19? If so, please share your experiences.

1. **COVID-19 and migration/movement**

F1. Have you or anyone in your household migrated or moved households as a result of conditions which have been influenced by the COVID-19 pandemic? Where and why did they move?

*Probe: as a result in increasing economic hardship, to search for work, to care for a sick family member, etc.*

*Probe: was this a move from a rural to urban community, or vice versa?*

*Probe: were any children married sooner than previously planned to alleviate economic hardships?*

1. **Conclusion**

F1. Has COVID-19 changed the way you interact with your community? How about other people in your household?

F2. Is there anything else you would like to share regarding your experiences during the COVID-19 pandemic?

F3. Do you have any questions for me about this research?
